# Spatial patterns of an endemic Mediterranean palm recolonizing old fields

**DOI:** 10.1002/ece3.2504

**Published:** 2016-11-09

**Authors:** Miguel E. Jácome‐Flores, Miguel Delibes, Thorsten Wiegand, José M. Fedriani

**Affiliations:** ^1^ Department of Conservation BiologyEstación Biológica de Doñana (EBD‐CSIC)SevilleSpain; ^2^ Department of Ecological ModellingHelmholtz Centre for Environmental Research GmbH ‐ UFZLeipzigGermany; ^3^ German Centre for Integrative Biodiversity Research (iDiv) Halle‐Jena‐LeipzigLeipzigGermany; ^4^ Technical University of LisbonInstitute of AgronomyCentre for Applied EcologyLisboaPortugal

**Keywords:** *C. humilis*, colonization front, endozoochorous seed dispersal, Mediterranean scrubland, plant size patterns, point pattern analysis, Thomas point process models

## Abstract

Throughout Europe, increased levels of land abandonment lead to (re)colonization of old lands by forests and shrublands. Very little is known about the spatial pattern of plants recolonizing such old fields. We mapped in two 21–22‐ha plots, located in the Doñana National Park (Spain), all adult individuals of the endozoochorous dwarf palm *Chamaerops humilis* **L**. and determined their sex and sizes. We used techniques of spatial point pattern analysis (SPPA) to precisely quantify the spatial structure of these *C. humilis* populations. The objective was to identify potential processes generating the patterns and their likely consequences on palm reproductive success. We used (1) Thomas point process models to describe the clustering of the populations, (2) random labeling to test the sexual spatial segregation, and (3) mark correlation functions to assess spatial structure in plant sizes. Plants in both plots showed two critical scales of clustering, with small clusters of a radius of 2.8–4 m nested within large clusters with 38–44 m radius. Additional to the clustered individuals, 11% and 27% of all *C. humilis* individuals belonged to a random pattern that was independently superimposed to the clustered pattern. The complex spatial pattern of *C. humilis* could be explained by the effect of different seed‐dispersers and predators' behavior and their relative abundances. Plant sexes had no spatial segregation. Plant sizes showed a spatial aggregation inside the clusters, with a decreasing correlation with distance. Clustering of *C. humilis* is strongly reliant on its seed dispersers and stressful environmental conditions. However, it seems that the spatial patterns and dispersal strategies of the dwarf palm make it a successful plant for new habitat colonization. Our results provide new information on the colonization ability of *C. humilis* and can help to develop management strategies to recover plant populations.

## 1 Introduction

The spatial distribution of plants is often codetermined by seed dispersal, and environmental and historical factors impinging on plant arrival, establishment, and survival (Castro, Figueroa, Muñoz‐Schick, & Jaksic, 2005). For instances, on endozoochore systems, the spatial pattern of adult plants should conserve signatures of the spatial patterning of seed dispersal (e.g., from highly scattered to highly aggregated), that is, strongly influenced by the disperser movements and spatial fecal marking behavior (Fedriani & Wiegand, 2014; Schupp, Jordano, & Gómez, 2010). Furthermore, the establishment of new individuals could be affected by local environmental conditions, such as moisture (Villers‐Ruiz, Trejo‐Vázquez, & López‐Blanco, 2003), soil type (Shaukat, Aziz, Ahmed, & Shahzad, 2012), rainfall (He et al., 2014), or temperature (Villers‐Ruiz et al., 2003). Also, human‐related historical factors of site management (e.g., plant removal, fire, cow grazing) provide another plausible explanation for certain spatial patterns (e.g., Camarero, Gutierrez, Fortin, & Ribbens, 2005). Therefore, detailed characterization of the spatial pattern of plant populations can help us to understand the mechanisms that created them (e.g., Fedriani et al., 2015; Wiegand, Gunatilleke, Gunatilleke, & Okuda, 2007).

The spatial distribution pattern of well‐established plant populations has been intensively investigated in both tropical and temperate habitats (Fedriani & Wiegand, 2014; Shaukat et al., 2012; Wiegand, Martínez, & Huth, 2009). However, very little is known about plant spatial patterns (and their causes and consequences) of plant populations recolonizing abandoned old fields (i.e., at their “colonization front”). This is somewhat surprising because environmental and socioeconomic changes are causing increased levels of land abandonment worldwide, leading to noticeable changes in landscape cover (Blondel, Aronson, Boudiou, & Boeuf, 2010; Cramer, Hobbs, & Standish, 2008; Thompson, 2005). This process is especially noticeable throughout the European backcountry, where forests and shrublands are spreading due to the decline of agricultural practices, pastoralism, and forest activities (Thompson, 2005). Thus, a better understanding of the plants spatial patterns and the underlying ecological mechanisms that create them is a prerequisite to understand the natural regeneration process and sustainable forest management.

Plants at a colonization front occur usually at low densities due to less favorable ecological conditions that reduce plant growth, survival, and reproduction, compared with large populations (Camarero et al., 2005; Chhin & Wang, 2002). For example, recolonizing populations may experience poorer plant and pollinator faunas (Stone & Jenkins, 2008), pollen limitation due to difficulty in finding a mate (Bessa‐Gomes, Clobert, Legendre, & Møller, 2003), limited seed dispersal (Holt, 2003), and inbreeding depression because of an insufficient number of founders (Ovaskainen & Hanski, 2001). In this context, we have a particular interest in characterizing quantitatively the spatial patterns of low‐density plant populations at their colonization fronts, as this can shed light about the ecological, environmental, and historical factors leading to it.

Spatial point pattern analysis (SPPA; Illian, Penttinen, Stoyan, & Stoyan, 2008; Wiegand & Moloney, 2014; Velázquez, Martínez, Getzin, Moloney, & Wiegand, 2016) comprises a suit of statistical techniques that allow for a detailed characterization of the smaller‐scale spatial distribution pattern of “ecological objects” such as plants. More generally, spatial point pattern data consist of the georeferenced locations (point) of every plant of a given type (e.g., adults) within a study plot, which can be supplemented by additional information characterizing the points (i.e., marks such as sex, size, or surviving vs. dead).

Of special interest in plant populations is to characterize the way they are spatially clustered. Thomas point processes are a class of relatively simple point process models that proved to be suitable for describing clustering in natural plant populations (e.g., Fedriani & Wiegand, 2014; Wiegand et al., 2009). In the simplest case of a Thomas process, the point pattern consists of a number of independently distributed clusters where the plants are scattered with a two‐dimensional normal distribution around the cluster centers. The parameters of the Thomas process can be fitted to the data, thereby providing a succinct description of the characteristics of the observed pattern, given a good fit (Wiegand et al., 2009), and allowing for insight into the processes that structure the populations. The Thomas process can also be extended to consider two critical scales of clustering that may be caused, for example, by two mechanisms of seed dispersal (Wiegand et al., 2009). It can also be extended to account for the presence of dispersers that produce different seed deposition patterns (e.g., scatter vs. clumped) that may lead to mixed patterns with a random component (due to scatter dispersal) and a clustered component (due to clumped dispersal; Wiegand et al., 2009; Fedriani, Wiegand, & Delibes, 2010). These extensions allow for a very realistic representation of more complex spatial patterns.

Marked SPPA techniques can be used to analyze the spatial correlation structure of plant traits (Fedriani et al., 2015; Illian et al., 2008). For example, in dioecious species, sexual spatial segregation (SSS) adds complexity to the spatial patterns and has been repeatedly observed in various species (e.g., Eppley, 2005). It has been described that SSS is generated by females preferring less stressful areas of the environment compared to males (Reuss‐Schmidt, Rosenstiel, Rogers, Simpson, & Eppley, 2015), to differential germination (Eppley, 2001), or to differential mortality between sexes (e.g., (Gibson & Menges, 1994). Finally, the sizes of plants are frequently spatially correlated. For example, Nakagawa, Yokozawa, and Hara (2015) found that aggregations are mostly composed of similar larger plants that compete and remove medium‐sized neighbors. Perturbation also affects neighboring plant populations; stressful habitats may show a bigger variety of plant sizes and ages compared to less perturbed areas (Weiner, Campbell, Pino, & Echarte, 2009).

In this study, we used SPPA to quantify the spatial distribution patterns of two low‐density populations of dwarf palm *Chamaerops humilis* L. that recolonized old fields in the Doñana National Park, Spain. The two populations differ in their management history that generated at one site a dehesa (i.e., grassland with scattered trees) and at the other site a dense Mediterranean scrubland. *C. humilis* is endemic to the Western Mediterranean basin and relatively abundant in Mediterranean scrub thickets and open pine forests, and endemic to the Western Mediterranean basin, with no obvious preference of type of soil or substratum (Herrera, 1989). Recently, anthropogenic pressures and the introduction of noxious pest have drastically reduced dwarf palm populations in part of their distribution range (Drescher & Dufaÿ, 2001; Rodríguez, Delibes, & Fedriani, 2014). A nearby control plot within similar habitat was not available. For this reason, we focussed on accurately describing the spatial patterns of two palm populations with different management history. Comparison of detailed characteristics of spatial patterns of the same species at two different sites will allow us to determine common drivers of the patterns and evaluate the impact of site differences.

More specifically, we tested the following three hypothesis: (1) Because both long‐ and short‐distance seed dispersers interact with *C. humilis* (Fedriani & Delibes, 2011), we expect that both populations will show a spatially aggregated pattern with several critical scales and possibly with a random component pattern; (2) the dwarf palms will not show SSS (i.e., the pattern of males or females will be a random subsample of the pattern of all individuals), due to equal dispersal mechanism and high tolerance of both sexes to stressful conditions; and (3) because plants in perturbed areas may show a large variety of sizes (Weiner et al., 2009), we expect that dwarf palms at both populations will have no spatial segregation by size.

## 2 Study species and area

### 2.1 Study species

**Figure 1:**
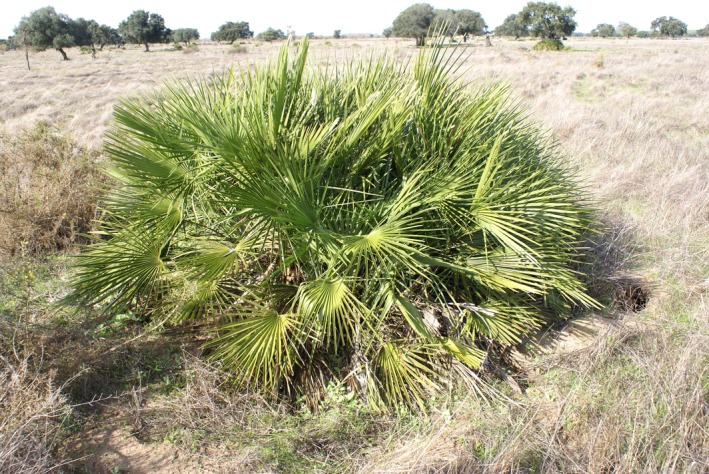
Adult plant of *C. humilis* at Doñana National Park

*C. humilis* is a small (usually ~1.5 m high; Figure 1) dioecious palm, considered a thermomediterranean bioindicator (Herrera, 1989). In Europe, it is usually not present beyond 1000 m above sea level, being most common in coastal areas. Due to its vigorous sprouting, *C. humilis* is very tolerant to disturbance (fire, herbivory, etc.; Herrera, 1989), and thus, it is often used in restoration programs in the context of global change (Rodríguez et al., 2014). It blooms during March–May, showing a mixed insect and wind pollination system (Anstett, 1999; Herrera, 1989). Specifically, its main pollinator seems to be the host‐specific palm flower weevil *Derelomus chamaeropsis* (Anstett, 1999). The fruits are “polydrupes,” comprising one to three drupes that ripe in autumn (September–November). Fruits are attached to infrutescences (or branches) of up to 30 cm long (37–91 fruits per branch).

In European populations, dispersal of *C. humilis* is almost exclusively by mammal species such as badgers (*Meles meles*), foxes (*Vulpes vulpes*), and rabbits (*Oryctolagus cuniculus*), but occasionally also by red deer (*Cervus elaphus*) (Fedriani & Delibes, 2011). Finally, seedlings emerge during the spring and the early summer, experiencing extensive mortality due to both summer droughts and herbivory (Fedriani & Delibes, 2009a,b). Although the dwarf palm has been described by Herrera (1989) as a plant with no obvious preferences regarding type of soil or substratum, we found that low areas and marshes susceptible to be flooded lack dwarf palms. In our study system, the Doñana Park, *C. humilis* is generally associated with sandy soils, presenting highly fragmented distributions due to both historical (crops, villages) and environmental (marshes, dune system) barriers.

### 2.2 Study area

**Figure 2:**
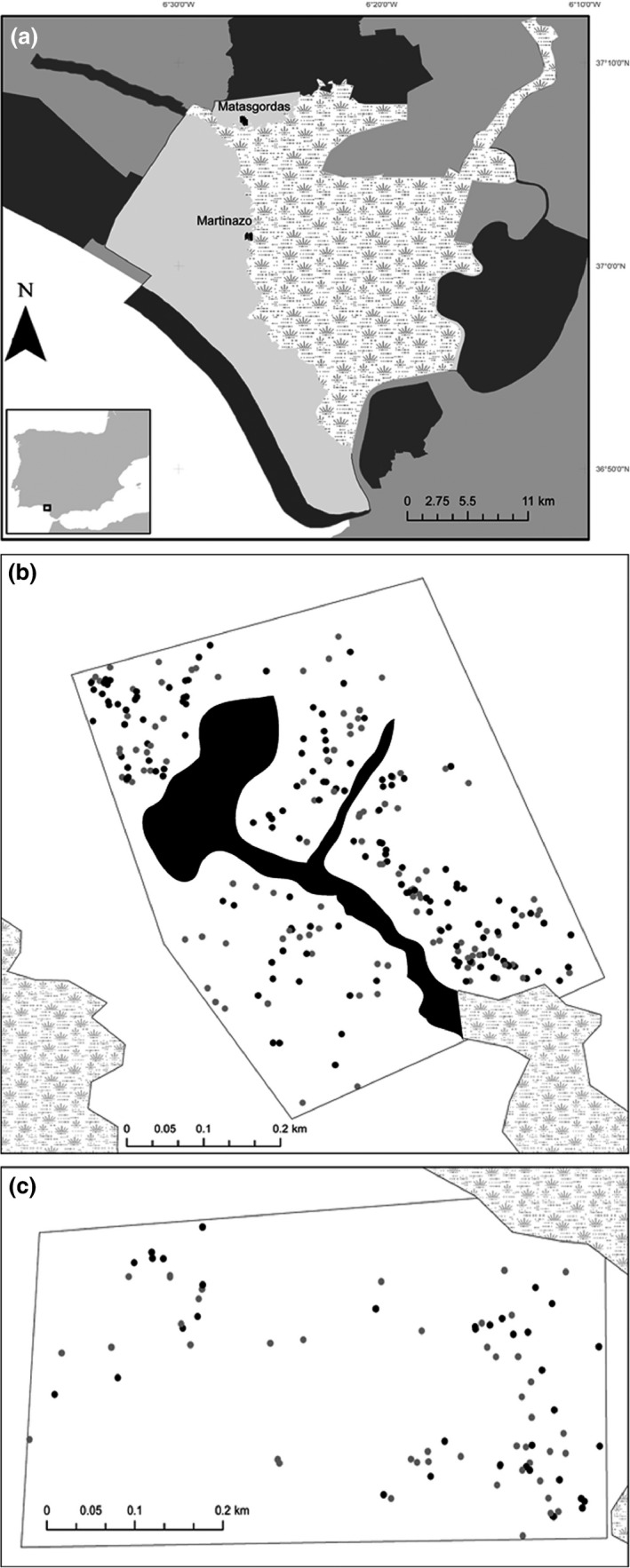
The study plots. (a) Location of two studies within the area of the Doñana National Park. The darkest gray represents the Doñana Nature Reserve, the medium grey nonprotected areas, the light gray the Doñana National Park, and the white area with grass pattern the marshland. (b) The Matasgordas plot with the georeferenced plants: The points in black represent female plants, and the gray points the males. We excluded an area inside the Matasgordas plot that was not suitable for the dwarf palm (black area) because of winter flooding

The study was carried out in the Doñana National Park (510 km^2^; 37°9′N, 6°26′W), located on the right bank of the Guadalquivir estuary in southwestern Spain. Average annual temperature ranges between 15.4 and 18.7°C (mean = 16.9 ± 1°C; *n* = 35; period 1978‐013). Annual rainfall was higly variable, ranging during this period between 170 and 1028 mm (mean = 542.6 ± 12 mm; data from Monitoring Team of Natural Process of Doñana Biological Station; http://www-rbd.ebd.csic.es/Seguimiento/mediofisico.htm). Most rain was concentrated from October to March. Between November and December of 2011, we selected and delimitated two observational plots within the Doñana area, called Matasgordas and Martinazo (Figure 2), where we identified and georeferenced (with a submetric GPS, accuracy = ±0.2 m) all adult reproductive *C. humilis* individuals (*n* = 399).

### 2.3 Study plots

The vegetation and physiographic characteristics differed between the two plots. The Matasgordas plot is occupied by a dehesa (i.e., grasslands with scattered trees), which includes some areas prone to flooding and is limited in the south by a marshland (Figure 2). The dehesa habitat (~300 ha) was generated in 1970 when all shrubs and most trees were mechanically removed. This management resulted in a continuum of grasslands with an open tree stratum of *Q. suber*,* O. europaea* var. *sylvestris*, and *Fraximus angustifolia* with no, or only sparse, understory of Mediterranean scrubs (Fedriani et al., 2010). The area was used for intensive cow grazing until 1996, when the land became owned by a governmental agency and the cows were removed, under the protection of the Spanish National Park Service. Since then, several mammal‐dispersed plants, including *C. humilis*, are recolonizing the area (Fedriani & Wiegand, 2014). In this site, we delimited a plot of 22.1 ha which included 308 adult individuals (Figure 2b).

The Martinazo site is covered by a dense Mediterranean scrubland dominated by *Halimium halimifolium*,* Rosmarinus officinalis*,* Ulex* spp., and *Stauracanthus* spp. Historically, this area has been used for hunting and livestock ranching, which increased the herbivory pressure over the native shrub. Additionally, woody species (*Quercus suber*,* Olea europaea* var. *sylvestris*) were cut down, and controlled rotating burnings were applied every 25–30 years (Granados, Martin, & García Novo, 1986, 1988). Since then, the area has been recolonized by vegetation composed mainly of a pyrophytic scrub (*Halimium* ssp, *Ulex* spp., *Stauracanthus* spp., and *C. humilis*) (Granados et al., 1988). Within the Martinazo site, we delimitated a plot of 21 ha which included 91 adult individuals of *C. humilis* (plants that had any flowering evidence) (Figure 2c). The extension of this plot was determined by the marshland in the northeast part (Figure 2c).

In both plots, we georeferenced all adult *C. humilis* individuals (Figure 2). For each plant, we registered the sex and the size. We identified the sex based in the inflorescences morphological differences: The female inflorescences are solid, brownish and had borne fruits or just the calyx; instead, the males have smaller and very brittle inflorescences. The size of each individual (projected area of the plant canopy) was approached as the area of an ellipse (range 0.06–15.8 m^2^), so we measured the minor and major radius.

### 2.4 Spatial pattern analysis

To address our three hypotheses, we conducted three types of analyses. *C. humilis* plants were clearly clustered (Figure 2). Thus, we used a sequence of Thomas cluster point process models with increasing complexity to characterize the observed plant clustering in detail. We used the random labeling null model to test whether female and male individuals were a nonrandom sample of all individuals as expected by SSS. Finally, we used mark correlation functions to investigate whether the sizes of all plants, or those of males and females, showed spatial correlations.

### 2.5 Analysis of clustering: summary functions

To comprehensively characterize the spatial patterns of the two *C. humilis* populations, we used four different summary functions: the pair correlation function *g*(*r*), the *L*‐function *L*(*r*), the spherical contact distribution *H*_s_(*r*), and the nearest neighbor distribution function *D*(*r*) (Illian et al., 2008; Wiegand & Moloney, 2014). Wiegand, He, and Hubbell (2013) showed that these summary statistics together are able to capture most of the potentially complex spatial structure of homogeneous patterns. The *g*(*r*) and *L*(*r*) can be calculated analytically for the Thomas cluster point processes used here and are therefore traditionally used to fit their parameters (e.g., (Diggle, 2003). The *H*_s_(*r*) and *D*(*r*) capture additional information that will allow us to find out whether the patterns were mixed patterns with a random component (e.g., due to scatter dispersal) and a clustered component (e.g., due to clumped dispersal) (see Wiegand et al., 2009).

For homogeneous patterns, the univariate pair correlation function *g*(*r*) can be defined as the density of points within a ring of radius *r* and width *dw* around the typical point of the pattern, divided by the intensity λ of the pattern (= the number of points divided by area). Thus, *g*(*r*) > 1 indicates clustering because the pattern shows a higher neighborhood density than expected by a random pattern (= λ). The *L*‐function is the transformation *L*(*r*) = (*K*(*r*)/π)^0.5^ − *r* of the *K*‐function which is the cumulative version of pair correlation function, that is, *K*(*r*) = ∫*g*(*r*) 2π *r* d*r*. For a random pattern, we find *L*(*r*) = 0, and for a clustered pattern *L*(*r*) > 0. While *L*(*r*) and *g*(*r*) are based on the same information, their joined use improved parameter fitting (Wiegand et al., 2009) because the pair correlation function is especially sensitive to clustering at small scales and the *L*‐function is more sensitive to clustering at larger scales.

The spherical contact distribution *H*_s_(*r*) yields the probability that a random “test” point has its first *C. humilis* neighbor at distance *r* and characterizes the “holes” in the pattern. If the pattern has a random component, the “holes” will be smaller than expected by the Thomas cluster process. Conversely, the nearest neighbor distribution function *D*(*r*) that characterizes the clustering of the *C. humilis* pattern returns the probability that the typical *C. humilis* individual has its first neighbor at distance *r*. If the pattern has a random component, the proportion of individuals that have their first neighbor at larger distances will be larger than expected by the Thomas cluster process.

### 2.6 Analysis of clustering: hypotheses

We used three cluster point process models with increasing complexity to characterize the properties of the clustering patterns in detail. In a first step, we tested whether a simple Thomas process that incorporated one critical scale of clustering (Wiegand, Gunatilleke, Gunatilleke, & Okuda, 2007) accurately described the spatial pattern of the two *C. humilis* populations. We used the pair correlation and the *L*‐function, which are known analytically for this Thomas process (equation (1)), to fit the two parameters using the minimum contrast method (Illian et al., 2008; : section 7.2.2) as described in Wiegand et al. (2009).

If the Thomas process with one critical scale of clustering did not fit the *g*(*r*) and *L*(*r*) well (especially if it underestimated the clustering at small scales), we used in a second step a more complex Thomas process that incorporates two critical scales of clustering. The four parameters of this point process were fitted again using the summary functions *g*(*r*) and *L*(*r*), which are analytically known for this point process (equation (2)), using the minimum contrast method in the sequential way described in Wiegand et al. (2009).

However, it is well known that the “second‐order” summary functions *g*(*r*) and *L*(*r*) do not fully determine a cluster point process (Diggle, 2003; Wiegand, Gunatilleke, Gunatilleke, & Okuda, 2007). For example, the analytical expressions for the pair correlation function (equations (1) and (2)) use only information on the expectation of *S*(*S *− 1), where *S* is the number of points of a cluster, but not on how the points are distributed over the clusters (see (Wiegand & Moloney, 2014): section 4.1.4). As a consequence, cluster processes with different distributions *p*_*S*_ of the number of points *S* per cluster, but the same expectation of *S*(*S *− 1) will show the same *g*(*r*) and *L*(*r*). To assess the distributions of the number of points per cluster, we therefore used the spherical contact distribution *H*_s_(*r*) and the nearest neighbor distribution function *D*(*r*) (Illian et al., 2008; Wiegand & Moloney, 2014). The distributions of the number of points per cluster are biologically of interest because it allows us to determine the proportion of “isolated” *C. humilis* individuals (a random component pattern) that may be created by a different seed disperser as the *C. humilis* individuals located in clusters. Thus, if the Thomas process with two critical scales of clustering (equation (2)) did not fit well the spherical contact distribution and the nearest neighbor distribution function (e.g., because the data contain more isolated *C. humilis* individuals than predicted by this cluster process; Wiegand et al., 2007), we used a point process that results from independent superposition of a Thomas process with two critical scales of clustering and a random pattern (equation (3)). The hypothesis is that the *C. humilis* individuals of the cluster component process were dispersed by a different agent than the *C. humilis* individuals of the random component process.

### 2.7 Analysis of clustering: point process models

The Thomas process with one critical scale of clustering consists of randomly and independently distributed “clusters” where ρ is the intensity of the cluster centers (i.e., the number of clusters divided by area). The points of the pattern are then randomly assigned to the clusters (i.e., the distribution *p*_*S*_ of the number of points *S* per cluster follows a Poisson distribution), and their distribution relative to the cluster center follows a two‐dimensional normal distribution with variance σ^2^. The cluster size *r*_C_ can be defined as *r*_C_ ≈ 2σ and includes approximately 87% of the points of a given cluster, and the approximate area covered by one cluster is *A*_C_ = π $r_{C}^{2}$ = 4πσ^2^ (Wiegand et al., 2009) The pair correlation function of this Thomas process is (Wiegand et al., 2009):

(1) $$g\left(r,\sigma ,\rho \right)=1+\frac{1}{\rho }\frac{\text{exp}(-r^{2}/4\sigma^{2})}{4\pi \sigma^{2}}$$

The Thomas process with two critical scales follows the same construction principle as the previous one with one critical scale of clustering. The only difference is that the cluster centers do not follow a random pattern, but are assumed to follow a Thomas process with one critical scale of clustering. This “double‐cluster” process has four unknown parameters: the intensities ρ_L_ and ρ_S_ of the centers of the large and small clusters, respectively, and the parameters σ_L_ and σ_S_ that define the size of the large and small clusters, respectively. Its pair correlation function yields (Wiegand et al., 2009):

(2) $$g\left(r,\sigma ,\rho \right)=1+\frac{1}{\rho_{S}}\frac{\text{exp}(-r^{2}/4\sigma_{S}^{2})}{4\pi \sigma_{S}^{2}}+\frac{1}{\rho_{L}}\frac{\text{exp}(-r^{2}/4\left(\sigma_{S}^{2}+\sigma_{L}^{2}\right))}{4\pi (\sigma_{S}^{2}+\sigma_{L}^{2})}$$

The third point process we used here is an independent superposition of a random pattern with a Thomas process with two critical scales of clustering (Wiegand, Gunatilleke, Gunatilleke, & Huth, 2007; Wiegand et al., 2009). In this point process, we first simulated the double‐cluster process described above, but only for *np*_C_ points, where *n* is the observed number of points of the pattern. In a second step, we independently placed the remaining *n*(1 − *p*_C_) points at random locations of the plot. The pair correlation function of this superposition process yields (Wiegand et al., 2009):

(3) $$g\left(r,\sigma ,\rho \right)=1+\frac{p_{C}^{2}}{\rho_{S}}\frac{\exp(-r^{2}/4\sigma_{S}^{2})}{4\pi \sigma_{S}^{2}}+\frac{p_{C}^{2}}{\rho_{L}}\frac{\exp(-r^{2}/4\left(\sigma_{S}^{2}+\sigma_{L}^{2}\right))}{4\pi (\sigma_{S}^{2}+\sigma_{L}^{2})}$$

Thus, the functional form of the pair correlation function is identical to that of the cluster process (2), but the number of clusters is virtually elevated by factor $1/p_{C}^{2}$. Thus, fitting the *g*(*r*) and *L*(*r*) cannot determine the additional parameter *p*_*C*_. However, superposition with a random pattern produces “isolated” points, which will affect both the shape of *H*_s_(*r*) and *D*(*r*). We therefore used the spherical contact distribution *H*_s_(*r*) and the nearest neighbor distribution function *D*(*r*) to determine the proportion *p*_C_ of random points. This was carried out by simulating the point process with several values of *p*_C_ and selecting the value of *p*_C_ that produced the best fit in *H*_s_(*r*) and *D*(*r*).

**Figure 3:**
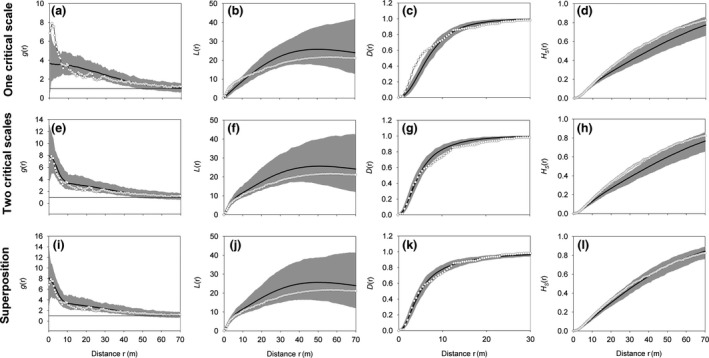
Cluster analysis of the Matasgordas plot. (a–d): fit with the Thomas process with one critical scale of clustering (equation (1)); (e–h): fit with Thomas process with two critical scales of clustering (equation (2)); and (i–l): fit with superposition of a random pattern with 30 points (*p*_C_ = 0.86) with a Thomas process with two critical scales of clustering (equation (3)). Open disks: observed summary functions, black line: expectation under the point process model, gray area: simulation envelopes being the fifth lowest and highest values of the summary functions estimated from the 199 simulations of the fitted cluster processes

Because the study sites showed irregular shapes (Figure 3b,c), we used the Ohser edge correction described in detail in Wiegand and Moloney (2014, equations 3.29 and 3.30) based on the isotropized set covariance for irregularly shaped study areas. To obtain a good resolution of the small‐scale clustering, we used a bin of 0.5 m and a ring width of 2.5 m for the estimation of the pair correlation function. The cluster processes are stochastic processes and that different realizations of the same cluster process will yield somewhat different patterns (as shown by the simulation envelopes of the simulated cluster processes). Thus, each realization generated with the same parameters would produce slightly different best‐fit parameters when fitted with the cluster process that generated the pattern.

To avoid overfitting, we took care that the best‐fitting simpler model caused clear departures from the observed summary functions. To this end, we estimated the standardized effect sizes (Getzin, Wiegand, & Hubbell, 2014; Punchi‐Manage et al., 2015; Velázquez et al., 2016)

(4) $$S_{0}^{\mathrm{ses}}\left(r\right)=\frac{S_{0}\left(r\right)-\overset{¯}{S}\left(r\right)}{{\hat{\sigma }}_{S}\left(r\right)},$$

of the original summary function *S*(*r*) where *S*_*i*_(*r*) are the summary functions estimated from the observed data (*i *=* *0) and from the *s* realizations of the null model (*i *=* *1,.. *s*), and $\overset{¯}{S}(r)$ and ${\hat{\sigma }}_{S}\left(r\right)$ are the mean and the standard deviation of the *S*_*i*_(*r*) estimated for *i *=* *1,.. *s*, respectively. Effect size $S_{0}^{\mathrm{ses}}$(*r*) >4 or <−4 can be regarded as clear departure from the point process model (Wiegand et al. 2016). Note that a significant departure at one fixed distance *r* with significance level of 0.05 occurs if the effect size is below −1.96 or above 1.96.

### 2.8 Random labeling

To test is the pattern of male and female *C. humilis* individuals were a random subsample of that of all individuals (i.e., the absence of spatial structure in the distribution of the two sexes), we contrasted the observed data with the random labeling null model that shuffles the labels “male” and “female” randomly over the dwarf palms (Wiegand & Moloney, 2014; : section 4.4.1). We used several test statistics based on pair correlation functions to test for departures from random labeling (Jacquemyn, Brys, Honnay, & Hutchings, 2009):

1. *p*_11_(*r*): tests if females show at distance r a pattern within all *C. humilis individuals*.
2. *p*_12_(*r*): tests if males at distance r are spatially associated with females
3. *dif*(*r*): tests if females are surrounded at distance *r* by a higher *C. humilis* density than males.

The test statistic *p*_11_(*r*) is the univariate mark connection function which yields the probability that two randomly selected dwarf palms that are at distance *r* apart both are females. The expectation of *p*_11_(*r*) under random labeling is *p*_11_(*r*) = $p_{1}^{2}$, where *p*_1_ is the proportion of females among all dwarf palms. For *p*_11_(*r*) > $p_{1}^{2}$, the females are clustered at distance *r* within all palms.

The test statistic *p*_12_(*r*) is a bivariate mark connection function, which yields the probability that two randomly selected dwarf palms, which are at distance *r,* the first is female and the second male. The expectation of *p*_12_(*r*) under random labeling yields *p*_12_(*r*) = *p*_1_ *p*_2_, where *p*_2_ is the proportion of males among all dwarf palms. For *p*_11_(*r*) < *p*_1_ *p*_2_, females and males are segregated at distance *r* within all palms.

Finally, the test statistic *dif*(*r*) compares the overall neighborhood density of dwarf palms at distance *r* around females with that around males and yields *dif*(*r*) = *g*_1,1+2_(*r*) − *g*_2,1+2_(*r*). If *dif*(*r*) > 0, females are located in areas of higher palm density than males.

### 2.9 Mark correlation function

To find out whether the sizes of both female and male individuals of *C. humilis* located distance *r* away were positively correlated, we used the framework of mark correlation function (Illian et al., 2008; Wiegand & Moloney, 2014: section 3.1.7). Our data comprise for each individual the coordinates, the sex (male or female), and the mark “size.” The bivariate mark correlation functions then consider all pairs of male and female palms (with index i, j and their marks *m*_i_ and *m*_j_, respectively), selects those pairs with interpoint distance *r*, and estimates the mean of a suitable test function *t*(*m*_i_, *m*_j_) over these pairs which is then divided by the expectation of the test function over all pairs *i*–*j*.

The *r*‐mark correlation function *k*_. m_(*r*) uses the test function

(5) $$t\left(m_{i},m_{j}\right)=m_{j},$$

and estimates therefore the mean size μ_f_(*r*) of females *j* that have a male *i* at distance *r*, divided by the mean size μ_f_ of all females, that is, *k*_. m_(*r*) = μ_f_(*r*)/μ_f_ (Wiegand & Moloney, 2014: section 3.1.7.5). Thus, *k*_. m_(*r*) > 1 indicates that females that have males at distance *r* are on average larger than expected. Conversely, *k*_m._ (*r*) < 1 indicates that females, which have males at distance *r*, are on average smaller than expected.

We are also interested in the correlation between the sizes of male and female palms that are distance *r* apart. The appropriate test function for this purpose was proposed by Schlather, Ribeiro, and Diggle (2004):

(6) $$t\left(r,m_{i},m_{j}\right)=\left[m_{i}-\mu_{m}\left(r\right)\right]\left[\left(m_{j}-\mu_{f}\left(r\right)\right)\right]$$

and results in a Morian's *I* like summary function *I*_mm_(*r*); this is a spatial variant of the classical Pearson correlation coefficient (Shimatani 2002) where μ_f_ and μ_m_ are the mean size of female and male dwarf palms, respectively. *I*_mm_(*r*) is normalized by σ_f_σ_m_ where $\sigma_{f}^{2}$ and $\sigma_{m}^{2}$ are the variances of the sizes of females and males, respectively.

To test whether male and female dwarf palms show nonrandom spatial correlations of their sizes, we contrasted the observed mark correlation functions to a null model that randomly shuffled the sizes within the female subpopulation and the male subpopulation, thus conserving the sex‐specific size structure (Wiegand, Raventós, Mújica, González, & Bonet, 2013; Wiegand & Moloney, 2014: section 3.1.7.5).

In all three analyses, we used 199 Monte Carlo simulations of the point processes and null models for construction of simulation envelopes, being the fifth highest and fifth lowest values of the summary function of the simulated patterns. If the observed summary function was inside the simulation envelopes, we considered the point process to satisfyingly describe the data. For all point pattern analyses, we used software *Programita* (Wiegand & Moloney, 2014) which can be accessed at www.programita.org.

## 3 Results

### 3.1 Dwarf palm spatial pattern

Dwarf palms at the Matasgordas plot showed two critical scales of clustering. Fit of the Thomas process with one critical scale of clustering (equation (1)) was not satisfying; the pair correlation function (Figures 2a and 3) and the *L*‐function (Figure 3b) were for distances below 4 m clearly outside the simulation envelopes of the fitted Thomas process. The effect sizes of the *g*(*r*) were for distances up to 4 m larger than four with a peak value of 5 at 3 m. This indicates that the data showed an additional small‐scale clustering not accommodated by this cluster process.

The best fit of the Thomas process with two critical scales of clustering (equation (2)) reveals approximately 19 large clusters with sizes 2σ_L_ = 38 m and approximately 401 small clusters with a size 2σ_S_ = 5.2 m which are nested within the previous 19 large clusters. This cluster process now fits the pair correlation function (Figure 3e) and the *L*‐function (Figure 3f) well. However, the observed nearest neighbor distribution function *D*(*r*) (Figure 3g) and the spherical contact distribution *H*_S_(*r*) (Figure 3h) are at some distances outside the simulation envelopes. Effect sizes for the *D*(*r*) were for distances above 15 m below values of −4. The observed *D*(*r*) is at distances above 7 m below the simulation envelopes, which indicates that the observed pattern contains more isolated points than the Thomas cluster process with two critical scales of clustering. Similarly, the observed *H*_s_(*r*) is above the simulation envelopes (i.e., the nearest neighbor of the test points is closer than predicted), which indicates that the holes in the observed pattern are smaller than those predicted by the Thomas cluster process. Thus, both the behaviors of *H*_s_(*r*) and *D*(*r*) indicate the existence of some isolated *C. humilis* individuals.

We simulated the superposition cluster processes (equation (3)) with different proportions 1–*p*_*C*_ of random points and found that a superposition cluster process with 30 random points (i.e., *p*_C_ = 0.89) yields simultaneous agreement in all four summary functions (Figure 3i–l). Thus, 11% of all *C. humilis* individuals may belong to the random component pattern. This point process showed a factor $p_{C}^{2}$ = 0.79, and therefore, 15 large clusters (= 0.79*19) and 317 small clusters (= 0.79*401) nested within the large clusters. Thus, each large cluster comprised on average 16.3 *C. humilis* individuals, and each small cluster on average 0.77 individuals. Because the number of points per small cluster follows a Poisson distribution with mean μ_s_ = 0.77, we can estimate for the cluster component pattern the expected number of small clusters with one individual (113), with two individuals (44), and more than two individual (14). Thus, approximately 52% of the *C. humilis* plants (30 + 113) had no nearby neighbor within the radius of the small clusters, but 32% had one neighbor and 16% had two or more.

**Figure 4:**
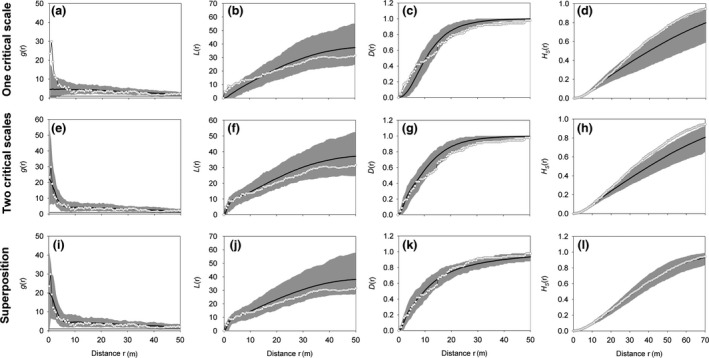
Same as Figure 3, but for the Martinazo plot

Results for the Martinazo plot were surprisingly similar to those of the Matasgordas plot. Again, the fit with the Thomas process with one critical scale of clustering revealed a signal in the data of an additional small‐scale clustering below 2 m (Figure 4a,b). The effect sizes of the *g*(*r*) were larger than 4 for distances below 2 m. Fit with the Thomas process with two critical scales of clustering reveals approximately 11 large clusters with an approximate radius of 2σ_L_ = 42 m and approximately 368 small clusters with a radius of 2σ_S_ = 2.8 m nested within the large clusters (Figure 4e). Again, this cluster process does not fit the nearest neighbor distribution function *D*(*r*) (Figure 4g) and the spherical contact distribution *H*_s_(*r*) (Figure 4h). However, here the departures were relatively weak with the effect sizes for the *D*(*r*) being below −3 for distances larger than 20 m. This is probably due to the lower sample size of this plot. Superposition with 25 random points (i.e., *p*_C_ = 0.73) yielded simultaneous agreement in all four summary functions (Figure 4i–l). This point process showed by factor $p_{C}^{2}$ = 0.53 and a reduced number of large and small clusters (i.e., six large clusters and 196 small clusters nested within the large clusters). Each large cluster comprised on average 11.3 individuals and each small cluster on average 0.34 individuals. Thus, 73 (= 48 + 25) all *C. humilis* individuals (79%) had no nearest neighbor within the radius of a small cluster, 17 (18%) one neighbor, and three (3%) more than one neighbor. This also shows that the small‐scale clustering at this site is weaker than that at the Matasgordas site (where approximately 52% of the *C. humilis* plants had no nearby neighbor within the radius of a small cluster).

### 3.2 The spatial relationship between females and males

**Figure 5:**
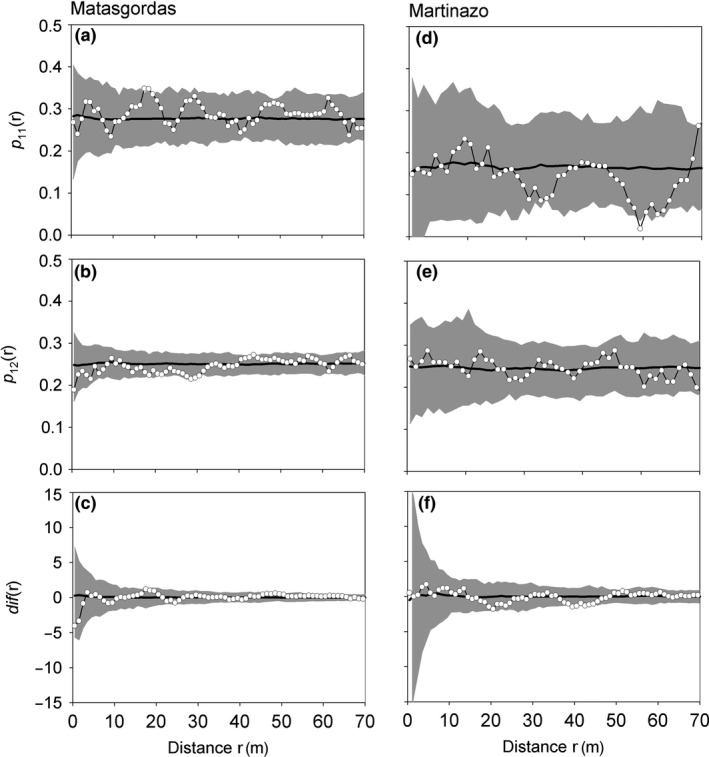
Results of the random labeling analysis to find out whether the distribution pattern of male and female dwarf palms was random within the overall pattern of all dwarf palms. (a–c) Results of the different test statistics for the Matasgordas plot, (d–f) results of the Martinazo plot. The *p*_11_(*r*) tests whether females show at distance *r* a pattern within all dwarf palms, the *p*_12_(*r*) tests whether males are at distance *r* spatially associated with females, conditionally on the locations of all dwarf palms, and the *dif*(*r*) tests whether females are surrounded at distance *r* by a higher dwarf palm density than males. The white circles represent the observed test statistics, the black line represents the expectation of the random labeling null model, and the gray area represents the simulation envelopes being the fifth lowest and highest values taken from 199 simulations of the null. We used a bin of 1 m and a ring width of 5 m

Our analysis using the random labeling null model showed that male and female dwarf palms in both observational plots did not show a spatial structure within all palms. Females were a random sample of all dwarf palms (Figure 5a,d), males were not segregated from females (Figure 5b,e), and the overall dwarf palm density around males and females did not differ (Figure 5c,f).

### 3.3 The spatial relationship among plant sizes

**Figure 6:**
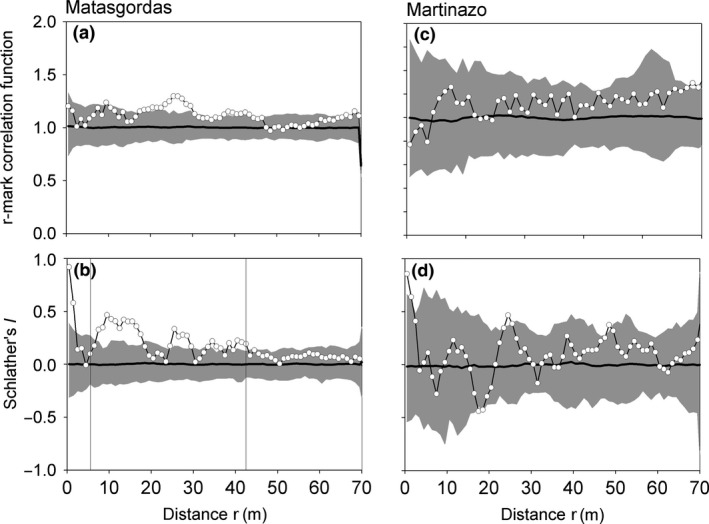
Results of the mark correlation analysis to find out whether the sizes of female individuals were correlated with those of male individuals at distance *r*. (a–b) Results for the Matasgordas site, (c–d) results of the Martinazo site. The r‐mark correlation function yields the mean size of females at distance *r* of males, and Schlather's I is the correlation coefficient between the sizes of all males and females separated by distance *r*. The white circles represent the observed summary functions, the black line represents the expectation of the random marking null model, and the gray area represents the simulation envelopes being the fifth lowest and highest values taken from 199 simulations of the null. We used a bin of 1 m and a ring width of 5 m (Matasgordas) and 7 m (Martinazo)

We found differences between the two populations. At Matasgordas, individuals that have another individual located within the range of large clusters (say 7–45 m) are larger than expected by the null model and individuals within the range of clustering (<45 m) show spatially correlated sizes (Figure 6a,c). There was also a very high correlation between plant sizes separated by distances below 3 m (this corresponds to the small clusters) and a moderate correlation over the range of the large‐scale clustering. This size correlation also appeared when we analyze separately females and males (Figures A1 and A2 in Appendix S1). In contrast, at the Martinazo plot we did not find significant values in the summary functions, so size of plants did not show a spatial pattern. (Figure 6b,d). This result is partly due to the smaller sample sizes, which produce substantially wider simulation envelopes.

## 4 Discussion

We analyzed the spatial patterns of adult dwarf palms in two areas of Doñana National Park (Spain) and found clearly identifiable spatial structures. The spatial patterns of the dwarf palms at the two contrasting study sites were structurally similar despite substantial differences in population density. Interestingly, cluster sizes and the random distribution of the sexes were very similar between the two populations. This suggests that the same underlying mechanisms may operate in both populations at their colonization front and generate similar spatial structures, which are then modified by different densities. This intriguing structural spatial similarity between both old fields let us to hypothesize that, if the disperser community would be similar, well‐preserved control plots would also show similar cluster sizes and a similar mixture of random and clustered components. However, because of its higher palm density the numbers of clusters (measured by the ρ_S_ and ρ_L_) and the number of palms per cluster (μ_S_ and μ_L_) should be higher in a control plot.

In nature, clustered patterns seem to be the rule (Wiegand, Gunatilleke, Gunatilleke, & Okuda, 2007), especially in plant populations dispersed by several frugivores with contrasting behaviors (e.g., Fedriani et al., 2010; Otero‐Arnaiz & Oyama, 2001). Fit with the complex Thomas process (equation (3)) revealed that the spatial pattern of the dwarf palm was characterized by a few large clusters (with radius of approximately 40 m) that hosted at Matasgordas and Martinazo averaging 16 and 11 individuals, respectively. Additionally, we found a small‐scale clustering where two or three palms sharing occasionally a small cluster with radius of some 5 and 3 m. Or in other words, in an average large cluster at Matasgordas with including 16 palms, eight have no nearby neighbor, but eight are arranged in groups of two or more. This grouping happened at Matasgordas and Martinazo for 48% and 21% of all palms, respectively. Additional to the clustered individuals, we estimated that 11% and 27% of all dwarf palms belonged to a random component pattern that was independently superimposed to the clustered component pattern. The existence of the two scales of clustering was clear for both plots, but due to relatively low sample size the random component pattern only weakly supported at the Martinazo plot. Thus, we have to interpret three features of the pattern: the random palms, the small‐scale aggregation, and the large‐scale aggregation.

Different behaviors of seed dispersers can impinge fruiting plant spatial patterns (Hampe et al. 2008). In the dwarf palm, there is a variety of seed dispersal agents, from occasional dispersers such as red deer (*C. elaphus*) to defleshers such as rabbits (*O. cuniculus*) and legitimate dispersers such as badgers (*M. meles*) and red foxes (*V. vulpes*) (Fedriani & Delibes, 2011). For instances, randomly distributed dwarf palms could be explained by the long‐distance dispersal carried out by the red fox, which deliver feces with seeds in a relatively scattered fashion (Fedriani et al., 2010). Also, the red deer (*C. elaphus*) and the wild boar (*Sus scrofa*), typically described as dwarf palm seed predators (Fedriani & Delibes, 2011), do allow some undamaged seeds to escape and fall randomly (authors' personal observation), contributing to the random plants in our plots. On the other hand, the existence of complementary dispersal mechanisms can explain dwarf palm aggregations. Large‐scale aggregations may be related to badger seed dispersal. Interestingly, although badgers act as long‐distance dispersers (Fedriani, Palomares, & Delibes, 1999; Revilla & Palomares, 2002), they tend to defecate dwarf palm seeds in large latrines at relatively small distance (~10 m) from the neighborhood plants (Fedriani & Wiegand, 2014), a fact that could increasing the size of plant patches. Besides, these large clusters were overlaid by small‐scale aggregations likely related to badger latrines, where feces containing seeds are strongly aggregated at small spatial scales, and to the vigorous sprouting (Fedriani & Delibes, 2011). Additionally, rabbits feed on ripe fruits, but they only eat the fleshy mesocarp, leaving the endocarp intact either still attached to ramets or detached and beneath mothers increasing plant recruitment at very short distances (Fedriani & Delibes, 2011). Our results are similar to those found for recruits of the tropical species *Shorea congestiflora* by Wiegand et al. (2007) and also tropical species *Cecropia insignis*,* Cordia bicolour*, and *Miconia argentea* by Wiegand et al. (2009). This suggests that complex double‐cluster and superposition patterns may be more common than previously thought.

Finally, the differences between sites could be explained by disparities in dispersers' activity. In Matasgordas, the density (and average activity) of badgers is higher (2.25 tracks km^−1^ day^−1^) than in Martinazo (1.46 tracks km^−1^ day^−1^) (data from Monitoring Team of Natural Process of Doñana Biological Station). Fedriani and Wiegand (2014) suggest that in areas with more badger activity seed aggregation should be higher, which subsequently may increase the number of aggregated palms. Instead, the density (and average activity) of foxes is higher in Martinazo (7.45 tracks km^−1^ day^−1^) than in Matasgordas (1.95 tracks km^−1^ day^−1^). The fox has a scattered seed dispersal pattern (Fedriani et al., 2010) that would generate a more sparse distribution, decreasing the percentage of aggregated plants in Martinazo. Furthermore, in Martinazo the herbivore pressure by native and domestic ungulates is high (Soriguer, 1983), eliminating most of dwarf palm seedlings and limiting plant aggregation.

Our results from the random labeling agree with our hypothesis that female and male palms did not differ in their spatial pattern within the study plots. Most dioecious species reflect SSS, generally correlated with environment or nutrient conditions, with males often in more nutrient‐poor or stressful environments than females (Eppley, 2005; Vessella, Salis, Scirè, & Piovesan, 2015). Nevertheless, we found that dwarf palm females and males did not differ in their spatial pattern, suggesting that there was no apparent microhabitat segregation by sexes. Furthermore, apparently dwarf palm females did not experience differential germination, differential mortality, or intrasexual competition, like in many other plant species with SSS (Eppley, 2001; Nanami, Kawaguchi, & Yamakura, 2005). The lack of differentiation between sexes in growth and survivorship was evident when we analyzed the palm sizes and did not find evidences of size differentiation. However, we found that plants in Matasgordas at least followed a size structure; in small and large aggregations, plants had similar sizes, with a decreasing positive autocorrelation with distance. This could be related to a facilitation process without intraspecific competition.

There are other factors that could affect the observed spatial patterns in our plots. As we described previously (see study area), both plots have been greatly affected by local human disturbances (e.g., livestock grazing), modifying and removing part of the original dwarf palm population (e.g., Thompson, 2005). In the last two decades, both plots have been slowly recolonized by pyrophytic shrub vegetation composed mainly of *H. halimifolium*,* R. officinalis*,* Ulex* spp., and *Stauracanthus* spp (Soriguer, 1983). This can explain the low density of dwarf palms in both plots (Matasgordas = 13.93 palms/ha; Martinazo = 4.34 palms/ha) compared with the ~400 palms/ha described in an unaltered close area (Fedriani & Delibes, 2011). The persistence of patches that are small or have low conspecific density is dependent on the successful reproduction of resident plants (Debinski & Holt, 2000; Groom, 2001). It is possible that the dwarf palm uses resident clustering as propagule sources to colonize new areas (Colautti, Grigorovich, & MacIsaac, 2006). Thus, the presence of two long‐distance dispersers (badgers and foxes) enables the colonization of new areas and increases plant density. When there is a significant long‐range dispersal, the edge of the range may extend some distance beyond the source population, forming a colonization front sustained by recurrent immigration (Pulliam, 1988). For instance, this could be happen in Matasgordas, where Fedriani et al. (1999, 2010) described that badgers deliver dwarf palm seeds to habitats where this palm is absent or occurs at low densities, like the dehesa. Finally, our study plots were in a transition zone of environmental stress, between the shrubland or dehesa and the marshland. Thus, species will expand their range to fill the available habitat until the plants reach marshland or zones with high‐flooding regime, where deterioration of the environment limits their survivorship (Drezner, 2014; Moore, 2009).

Populations with low density may experience lower reproductive output than their conspecifics in large populations (e.g., Allee effect; Ågren, Ehrlén, & Solbreck, 2008; Fedriani et al., 2015). Plants in small patches can be affected in their pollination regimes, being less attractive to pollinators (Fagan et al., 2014) or cause inbreeding depression because of an insufficient number of individuals (Ovaskainen & Hanski, 2001). However, several studies have shown that some species do no present lower fitness in marginal populations (e.g., Kluth & Bruelheide, 2005; Samis & Eckert, 2007), suggesting that they may have other mechanisms to maintain itself. For instance, in dwarf palm contagious occurrence of large individuals of both sexes, which tend to have more flowering resources than smaller ones (Méndez & Karlsson, 2004), can report obvious benefits in terms of pollination success (Fedriani & Delibes, 2009a; Gascoigne, Berec, Gregory, & Courchamp, 2009). Additionally, dwarf palm aggregations may result in potential benefits on fruit removal and dispersal (Carlo & Morales, 2008; Fedriani et al., 2010; Saracco, Collazo, Groom, & Carlo, 2005). In another study, we have observed not only higher seed dispersal, but also higher seed predation, in more aggregated plants (M. E. Jácome‐Flores, M. Delibes & J. M. Fedriani, unpublished). Spatial aggregation can negatively affect palm reproductive performance by attracting more seed and seedling predators (Fedriani & Delibes, 2011; Rodríguez et al., 2014). This is related to the Janzen–Connell hypothesis, according to which seeds and young plants would suffer increased mortality in the neighborhood of their parent plants (Connell, 1971; Janzen, 1970). Several studies have shown that survival of seedlings decreases with increasing density of conspecific seedlings and adults (Harms, Wright, Calderón, Hernández, & Herre, 2000; Metz, Sousa, & Valencia, 2010). However, Fedriani and Delibes (2011) frequently observed *C. humilis* seedlings establishing beneath fruiting palms, which suggest that the clumped pattern could “protect” seedlings from drought (e.g., Montesinos, de Luís, Verdú, Raventós, & García‐Fayos, 2006).

## 5 Conclusion

Spatial point pattern analysis provides a detailed description of the spatial structure of *C. humilis* populations recolonizing old fields and led us to infer some of the underlying processes generating these patterns. Additionally, this allowed us to make predictions on the spatial structure of healthy *C. humilis* populations and on the consequences for the palm reproductive performance and fitness. For example, the adult plants located in clusters could attract more pollinators and seed dispersers and predators, and could act as a nursery plant for its seedlings. It seems that the dwarf palm spatial patterns and dispersal strategies make from this species a successful plant for colonization of new habitats. Furthermore, it is a very tolerant species well adapted to high temperatures and intense droughts making a desirable species in restoration programs in the context of global change (Rodríguez et al., 2014). Clearly, to guarantee the restoration with this species, seedlings must be assembled in clumps of contrasting sizes and domestic ungulates must be removed from the area to accelerate colonization. Additional work is currently underway focusing on the effects of the dwarf spatial patterns over pollination and seed dispersal success, predation, and seedling survivorship. These data should help to assess the colonization ability of the dwarf palm and to develop management strategies to recover plant populations.

## Conflict of Interest

None declared.

## Data Accessibility

http://purl.org/phylo/treebase/phylows/study/TB2:S18857?x-access-code=689f72a1a127e905a1a46885c57480d2&format=html.
