# Supplementary material for: Spatial patterns of an endemic Mediterranean palm recolonizing old fields
Source: Ecol Evol. 2016 Nov 9;6(23):8556–68. doi: 10.1002/ece3.2504 (PMC5167057; doi:10.1002/ece3.2504)
Supplement: Supplementary file 1 [file ECE3-6-8556-s001.docx]

***Appendix 1***

|  |
| --- |
| **Figure A1.** Same as Fig. 5, but for the pattern of females and males. Individuals that have another individual located within the range of large clusters (say 7-45m) show are larger than expected by the null model and individuals within the range of clustering (<45m) show spatially correlated sizes. All summary statistics in Matasgordas are significant. The ring width was 5m for Matasgordas and 7m for Martinazo. |

|  |
| --- |
| **Figure A2.** Same as Fig. 5, but using the random labeling null model that maintains the sizes of the individuals but randomly shuffles their label “male” and “female”. The ring width was 7m for Matasgordas and 9m for Martinazo. Females at distance *r* away from males tend to be slightly larger than expected at the Matasgordas plot. |
