# Supplementary figures and images for: Spatial patterns of an endemic Mediterranean palm recolonizing old fields
Source: Ecol Evol. 2016 Nov 9;6(23):8556–68. doi: 10.1002/ece3.2504 (PMC5167057; doi:10.1002/ece3.2504)

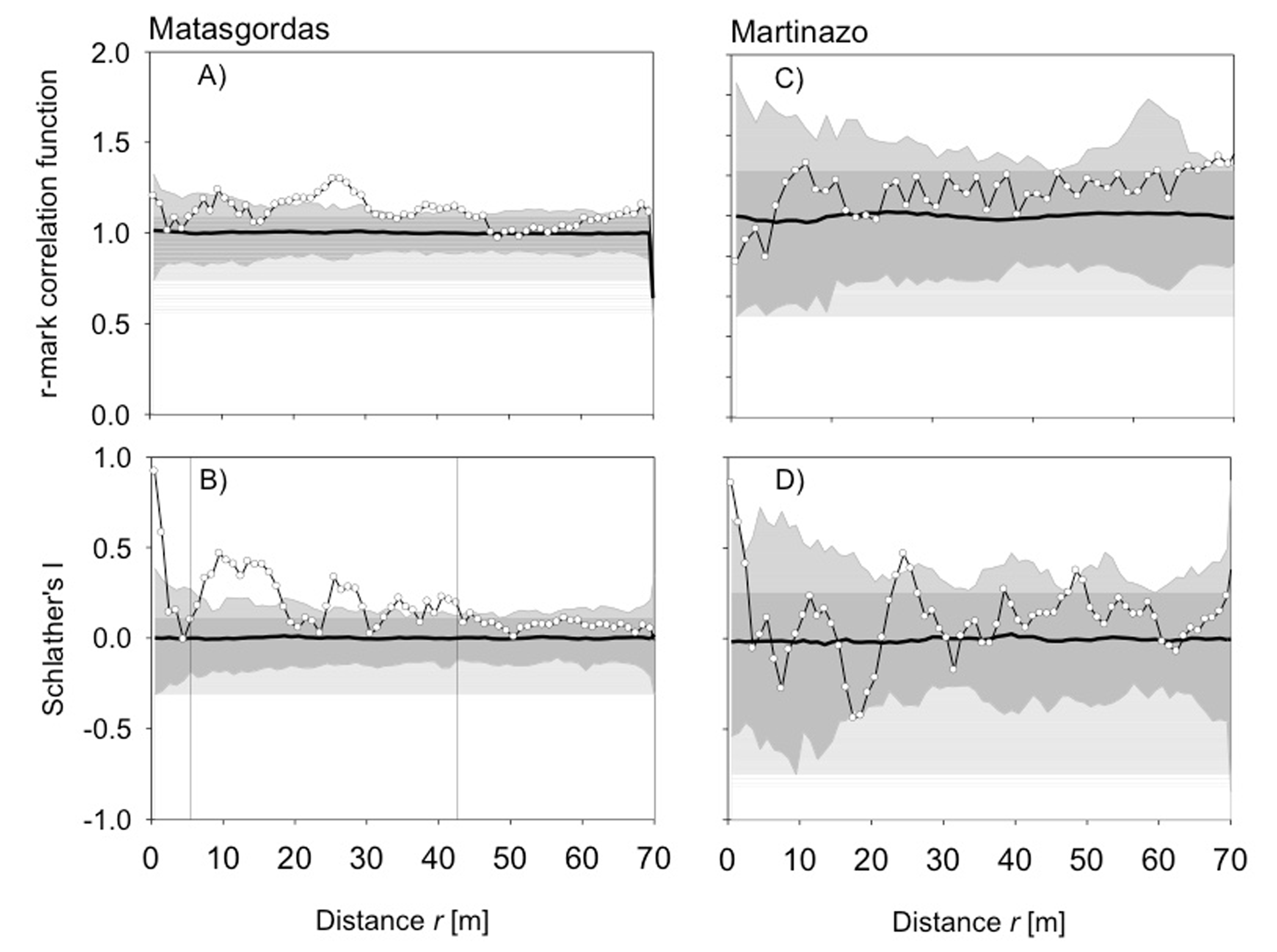

Supplement: Supplementary file 2 [file ECE3-6-8556-s002.jpg]

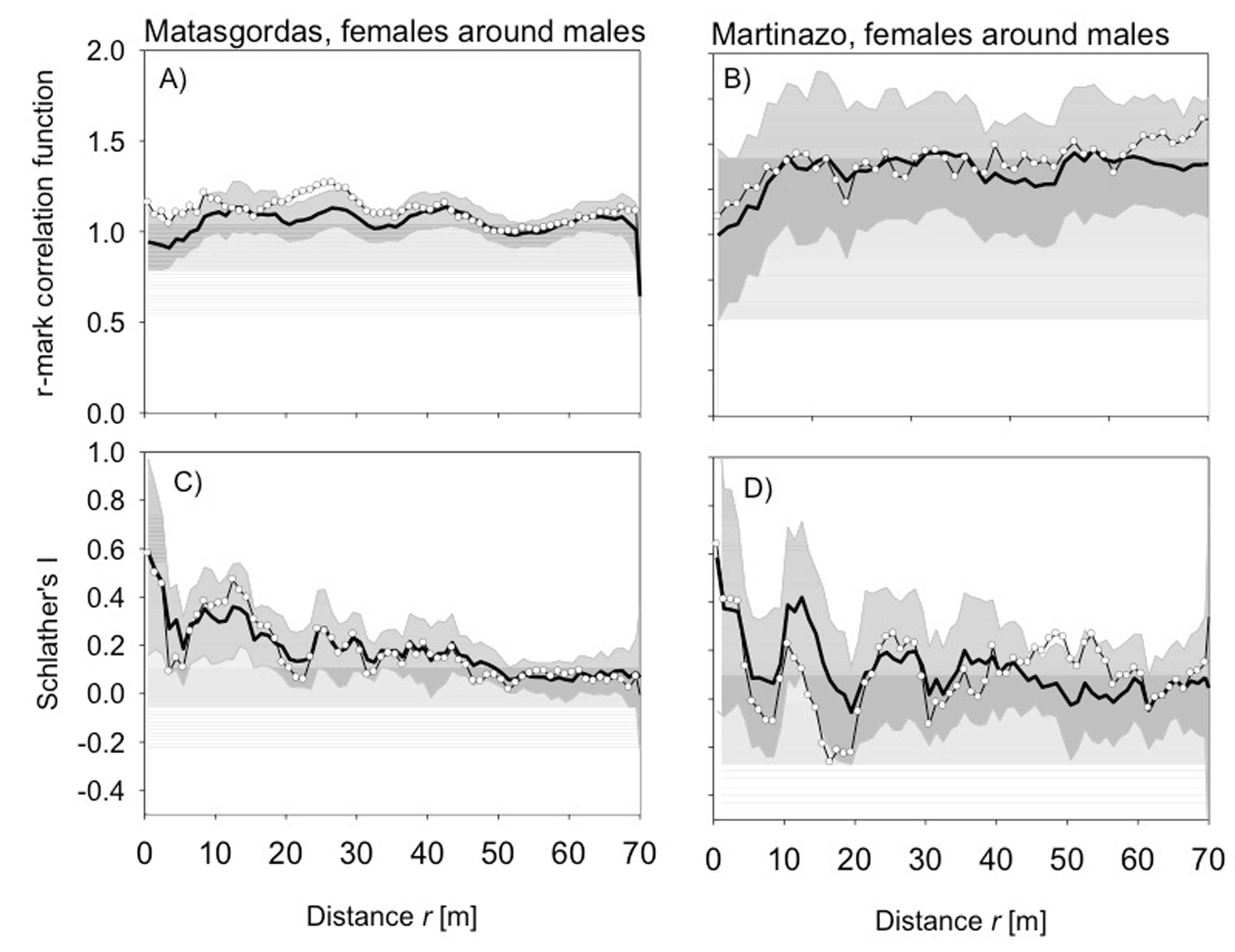

Supplement: Supplementary file 3 [file ECE3-6-8556-s003.jpg]
